# Research landscape and trends of cerebral amyloid angiopathy: a 25-year scientometric analysis

**DOI:** 10.3389/fneur.2023.1334360

**Published:** 2024-01-08

**Authors:** Kunyu Wang, Beilin Zhang, Heqian Du, Hanying Duan, Zhuoya Jiang, Shaokuan Fang

**Affiliations:** Department of Neurology, Neuroscience Research Center, The First Hospital of Jilin University, Changchun, China

**Keywords:** cerebral amyloid angiopathy, scientometric analysis, VOSviewer, CiteSpace, trends

## Abstract

**Background:**

Cerebral amyloid angiopathy (CAA), a cerebral small vessel disease affecting leptomeningeal and cortical small blood vessels, is a common cause of spontaneous lobar intracerebral hemorrhage and cognitive impairment, particularly in elderly patients. This study aims to investigate the field of CAA research from a scientometric perspective.

**Methods:**

Publications related to CAA from January 1st, 1999 to September 29th, 2023 were retrieved from the Web of Science Core Collection database. The scientometric software VOSviewer and CiteSpace were used to analyze and visualize the publication trends, countries/regions, institutions, authors, journals, cited references, and keywords of CAA.

**Results:**

A total of 2,798 publications related to CAA from 73 countries/regions, led by the United States, were included. The number of publications showed an increasing trend over time. Massachusetts General Hospital was the most productive institution, and authors Greenberg and Charidimou published the most papers and were most frequently co-cited. *Journal of Alzheimer's Disease* was the most prolific journal in this field, and *Neurology* was the most co-cited journal. Apart from “cerebral amyloid angiopathy”, the most frequently used keywords were “Alzheimer's disease”, “amyloid beta”, “intracerebral hemorrhage”, and “dementia”. The burst keywords in recent years included “cortical superficial siderosis” and “dysfunction”.

**Conclusions:**

This scientometric analysis provides a comprehensive overview of CAA research over the past 25 years, and offers important insights for future research directions and scientific decision-making in this field.

## 1 Introduction

Cerebral amyloid angiopathy (CAA) is a cerebral small vessel disease characterized by the accumulation of amyloid protein in the walls of leptomeningeal and cortical small blood vessels, primarily affecting arterioles and capillaries, occasionally involving venules (1, 2). Several types of amyloid protein have been found to be associated with CAA, among which the most common is amyloid β-protein (Aβ) (2). CAA can be categorized into three etiological types: sporadic, hereditary, and iatrogenic, with sporadic CAA being the most prevalent, while iatrogenic CAA is a newly proposed concept (3). Despite numerous *in vivo* and *in vitro* studies, the pathogenesis of CAA remains incompletely understood. Pathological changes secondary to Aβ deposition can lead to vascular wall rupture, resulting in spontaneous intracerebral hemorrhage (ICH), cerebral microbleeds (CMBs), convexity subarachnoid hemorrhage (cSAH) and the subsequent formation of cortical superficial siderosis (cSS), as well as ischemic changes such as white matter hyperintensities (WMHs) and cortical microinfarcts (1, 2, 4). CAA is also a significant cause of cognitive impairment in the elderly. Unlike other neurological disorders, the understanding of CAA started with pathologists, and it was not until the correlation between CAA and cerebral lobar hemorrhage was discovered that the disease received attention from neurologists (5). Advancements in neuroimaging techniques have made non-invasive diagnosis of CAA possible, and the Boston criteria based on imaging findings have been extensively validated (6). Molecular and functional imaging techniques have also provided new ways for a deeper understanding of CAA (7). Currently, there is no therapeutic interventions to alter the natural course of CAA, and disease-modifying treatments are still in the exploratory stage (8). The management of CAA patients primarily focuses on reducing the risk of hemorrhage (8).

Over the past 25 years, numerous studies on CAA have been published. However, it is challenging for researchers to identify highly influential articles and research hotpots. Scientometric analysis is an emerging approach that utilizes statistical methods and visualization to rapidly explore the structure and trends of a topic (9, 10). It encompasses quantitative and qualitative analyses that disclose various publication characteristics, such as identifying countries, institutions, authors, and journals contributing to a specific research field, highlighting frequently cited studies and commonly utilized keywords, and building the collaborations between countries, institutions, and authors (10). Therefore, scientometric analysis conveniently provides an understanding of the development and frontiers of a particular research field for new researchers. Scientometric analysis has been applied widely in a variety of medical fields, such as psychiatry (11, 12), oncology (13), infectious diseases (14), nursing (15), and neurology (16, 17).

The field of CAA research has experienced significant growth, leading to a consistent rise in the number of publications in this area. However, there is a notable absence of a comprehensive scientometric analysis that encompasses the latest research on CAA. To address this gap, we conducted a study utilizing two widely recognized scientometric software, VOSviewer (18) and CiteSpace (19), to conduct a thorough scientometric analysis of CAA studies published between 1999 and 2023. The primary objective of this article is to identify pivotal evidence and shed light on emerging trends in CAA research.

## 2 Materials and methods

### 2.1 Data collection

**Figure 1:**
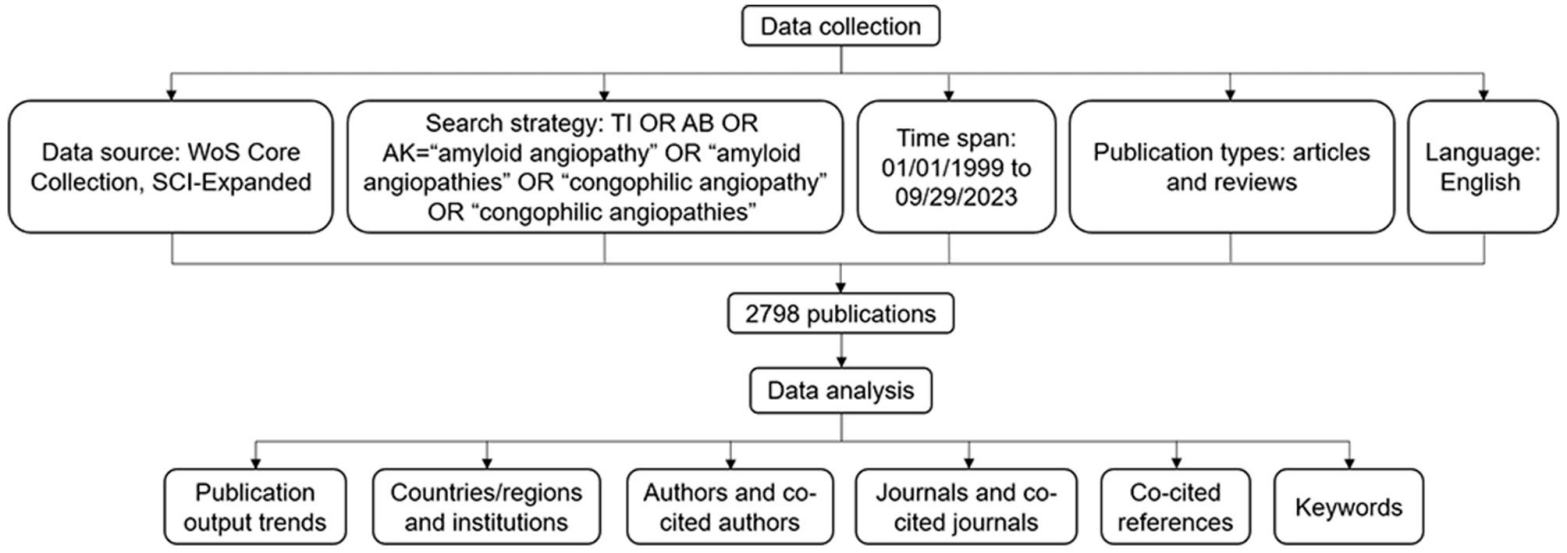
Flowchart of data collection and analysis.

The data for this study were obtained from Science Citation Index Expanded (SCI-E), Web of Science Core Collection (WoSCC). To avoid possible bias due to continuous database updates, all data were retrieved on September 30th, 2023. Relevant publications were collected using the following search formula: #1: TI=(“amyloid angiopathy” OR “amyloid angiopathies” OR “congophilic angiopathy” OR “congophilic angiopathies”); #2: AB=(“amyloid angiopathy” OR “amyloid angiopathies” OR “congophilic angiopathy” OR “congophilic angiopathies”); #3: AK=(“amyloid angiopathy” OR “amyloid angiopathies” OR “congophilic angiopathy” OR “congophilic angiopathies”); #4: #1 OR #2 OR #3. Publication types were limited to articles and reviews, excluding non-English literature. The period was set from January 1st, 1999 to September 29th, 2023. The detailed search and analysis procedure is shown in Figure 1.

### 2.2 Data analysis

All included papers from WoSCC were imported into VOSviewer and CiteSpace software for the purposes of analysis and visualization. VOSviewer (V.1.6.19) (Leiden University, Leiden, the Netherlands) was employed to perform visualization analysis of countries/regions and institutions, authors and co-cited authors, as well as journals and co-cited journals. CiteSpace (6.2.R4) (Chaomei Chen, Philadelphia, Pennsylvania, USA) was utilized to summarize the number of papers published each year, conduct visualization analysis of co-cited references and keywords, and generate dual-map overlay of journals.

## 3 Results

### 3.1 Analysis of publication output trends

**Figure 2:**
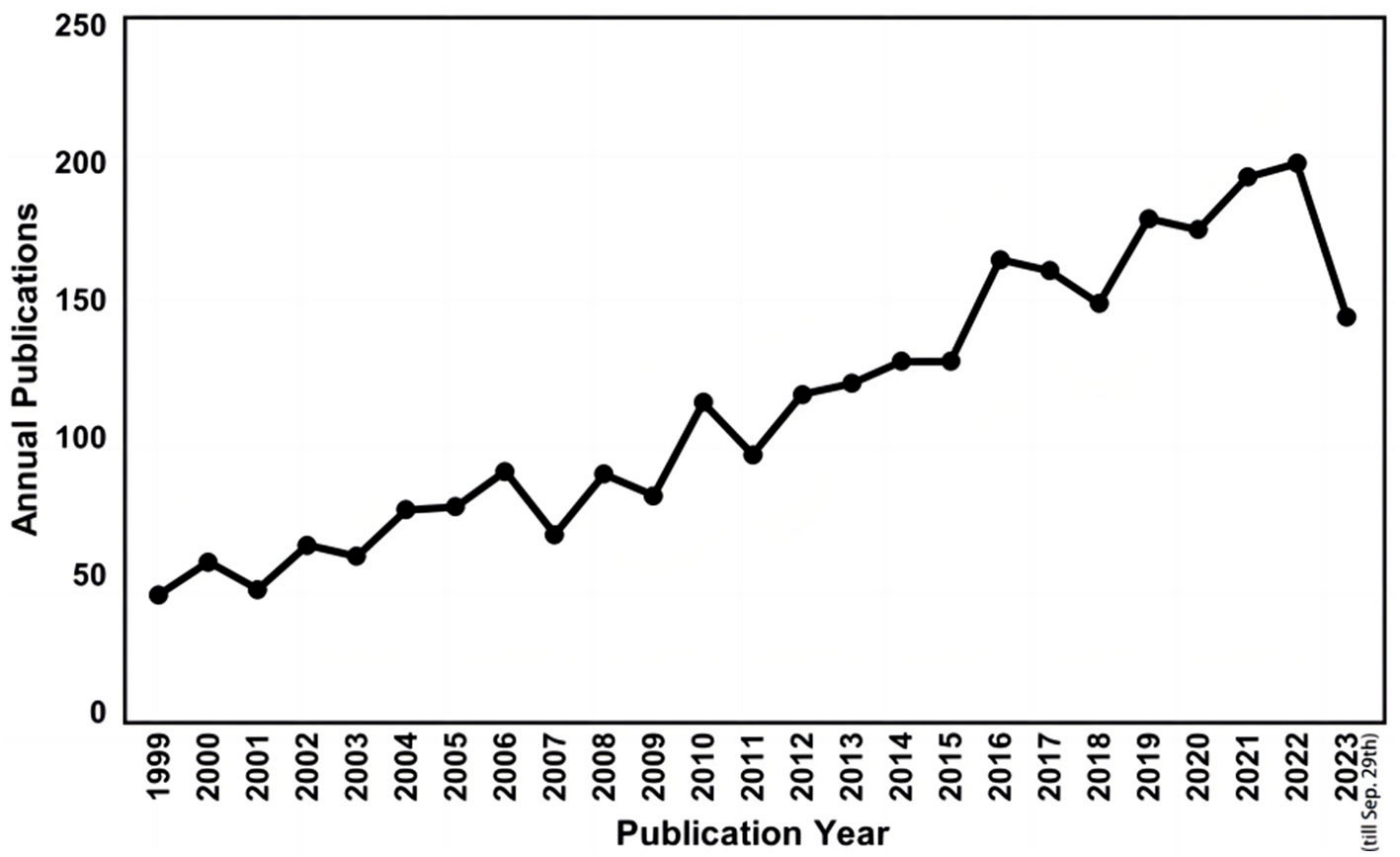
Trends of publications on CAA from 1999 to 2023.

A total of 2,798 papers (2,377 articles and 421 reviews) directly or indirectly related to CAA, published between 1999 and 2023 (till September 29th), were included in this study. The dynamic changes in the number of publications reflected the general development trend in the field. From 1999 to 2023, the trend line of annual publications was generally on the rise, and reached the peak in 2022 with 200 publications (Figure 2). These trends indicate that the research of CAA is flourishing and has gradually attracted the attention of researchers.

### 3.2 Analysis of countries/regions and institutions

**Figure 3:**
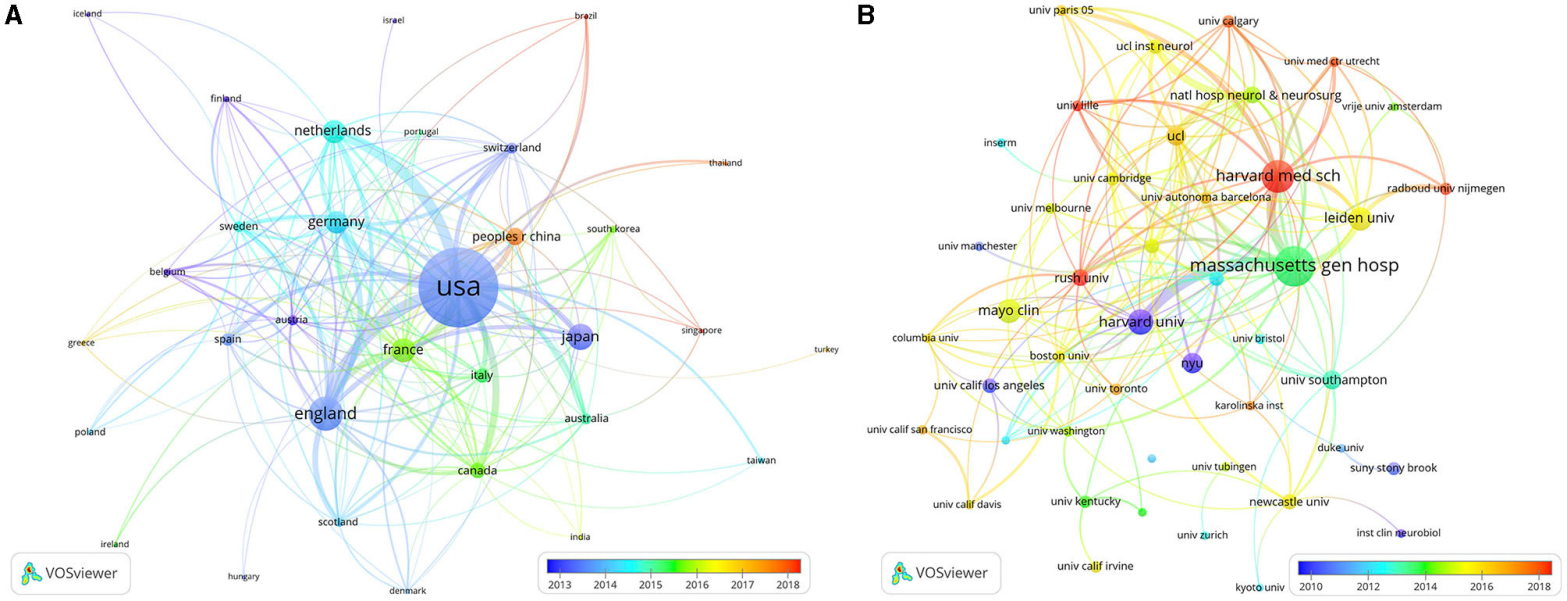
The visualization network of countries/regions **(A)** and institutions **(B)** on research of CAA.

These publications originated from 73 countries/regions and 2,679 institutions. The top 10 countries/regions were distributed in Europe, Asia, and North America, mainly in Europe (Table 1). The country/region with the highest number of publications was the United States (*n* = 1,246, 44.5%), followed by England (*n* = 404, 14.4%), Japan (*n* = 281, 10.0%), and France (*n* = 250, 8.9%). Over the past 25 years, the United States has been involved in nearly half of the CAA research. In addition, we filtered and visualized the countries/regions with a publication count equal to or greater than 10, constructing a collaborative network on the basis of the number and relationships of publications in each country/region (Figure 3A). There were extensive collaborations among different countries/regions. For instance, the United States exhibited close cooperation with nearly all countries/regions in the network; Japan actively collaborated with England, the Netherlands, Germany, and China.

**Table 1:** Top 10 countries/regions and institutions on research of CAA.

**Rank**	**Country/region (continent)**	**Count (%)**	**Institution (country/region)**	**Count (%)**
1	The United States (North America)	1,246 (44.5%)	Massachusetts General Hospital (The United States)	200 (7.1%)
2	England (Europe)	404 (14.4%)	Harvard Medical School (The United States)	148 (5.3%)
3	Japan (Asia)	281 (10.0%)	Harvard University (The United States)	106 (3.8%)
4	France (Europe)	250 (8.9%)	Mayo Clinic (The United States)	97 (3.5%)
5	The Netherlands (Europe)	241 (8.6%)	Leiden University (Netherlands)	94 (3.4%)
6	Germany (Europe)	233 (8.3%)	New York University (The United States)	79 (2.8%)
7	China (Asia)	166 (5.9%)	University College London (England)	77 (2.8%)
8	Canada (North America)	144 (5.1%)	University of Southampton (England)	72 (2.6%)
9	Italy (Europe)	129 (4.6%)	National Hospital for Neurology and Neurosurgery (England)	63 (2.3%)
10	Spain (Europe)	110 (3.9%)	Rush University (The United States)	62 (2.2%)

The top 10 institutions were distributed in 3 countries/regions, with 6 of them distributed in the United States (Table 1). The 3 institutions that published the most germane papers were: Massachusetts General Hospital (*n* = 200, 7.1%), Harvard Medical School (*n* = 148, 5.3%), and Harvard University (*n* = 106, 3.8%), highlighting the leading position of Harvard University and its affiliated institutions in CAA research. Subsequently, we selected institutions with a minimum publication count of 25 for visualization, and established a collaborative network on the basis of the number and relationships of publications of each institution (Figure 3B). As depicted in Figure 3B, there was significant collaboration among the top 10 institutions.

### 3.3 Analysis of authors and co-cited authors

**Figure 4:**
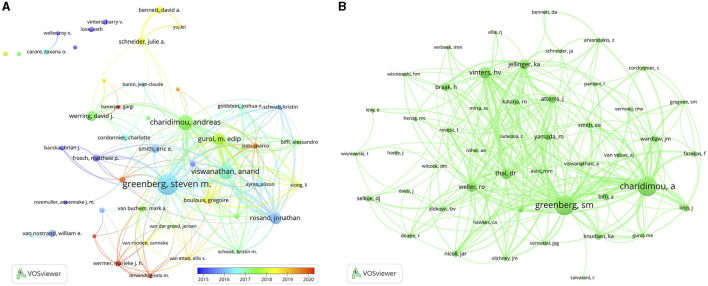
The visualization network of authors **(A)** and co-cited authors **(B)** on research of CAA.

A total of 12,917 authors have made contributions to CAA research. Three authors out of the top 15 published more than 100 papers each (Table 2). We created a collaborative network according to authors with a publication count equal to or greater than 19 (Figure 4A). Greenberg, Charidimou, and Viswanathan had the largest nodes due to their extensive publications. Furthermore, we noticed close collaborations among numerous authors. For example, Greenberg, Charidimou, Viswanathan, Gurol, Rosand, Smith, and Frosch exhibited a strong collaborative relationship.

**Table 2:** Top 15 authors and co-cited authors on research of CAA.

**Rank**	**Author**	**Count (%)**	**Co-cited author**	**Co-citation**
1	Greenberg, Steven M.	173 (6.2%)	Greenberg, SM	1,845
2	Charidimou, Andreas	109 (3.9%)	Charidimou, A	1,788
3	Viswanathan, Anand	109 (3.9%)	Vinters, HV	918
4	Gurol, M. Edip	78 (2.8%)	Thal, DR	911
5	Rosand, Jonathan	75 (2.7%)	Weller, RO	746
6	Werring, David J.	65 (2.3%)	Jellinger, KA	725
7	Smith, Eric E.	48 (1.7%)	Attems, J	607
8	Schneider, Julie A.	44 (1.6%)	Braak, H	572
9	Frosch, Matthew P.	41 (1.5%)	Yamada, M	532
10	Bennett, David A.	39 (1.4%)	Linn, J	494
11	Van Nostrand, William E.	39 (1.4%)	Wardlaw, JM	469
12	Boulouis, Gregoire	38 (1.4%)	Kalaria, RN	436
13	Cordonnier, Charlotte	37 (1.3%)	Knudsen, KA	431
14	Van Buchem, Mark A.	34 (1.2%)	Biffi, A	425
15	Van Veluw, Susanne J.	34 (1.2%)	Nicoll, JAR	418

Among the 36,861 co-cited authors, 9 had a co-citation frequency exceeding 500 (Table 2). It is noteworthy that Greenberg and Charidimou not only had the maximum quantity of publications, but also the maximum quantity of co-citations. We filtered authors with a minimum co-citation count of 200 to generate a co-citation network graph (Figure 4B). As depicted in Figure 4B, there were active co-citation relationships among authors, such as Greenberg, Charidimou, Vinters, Thal, and Weller.

### 3.4 Analysis of journals and co-cited journals

**Figure 5:**
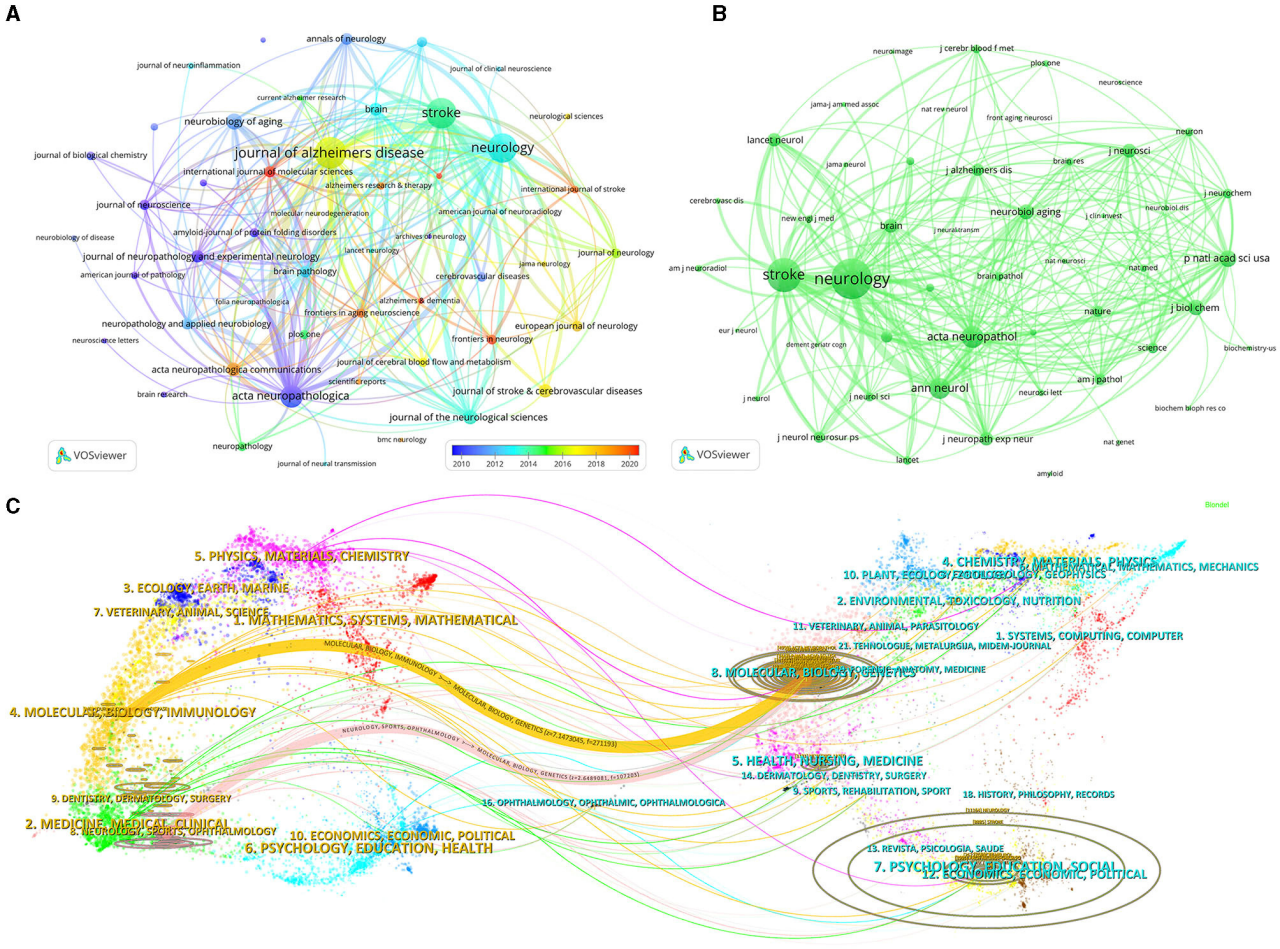
The visualization network of journals **(A)** and co-cited journals **(B)** on research of CAA. **(C)** The dual-map overlay of journals on research of CAA.

Publications relevant to CAA were published in a total of 542 different journals, with 3 of them publishing more than 100 papers each (Table 3). The top 10 prolific journals had impact factors (IFs) ranging from 2.5 to 14.5. The journal with the highest number of outputs on CAA was *Journal of Alzheimer's Disease* (*n* = 148, 5.3%), followed by *Stroke* (*n* = 141, 5.0%), *Neurology* (*n* = 136, 4.9%) and *Acta Neuropathologica* (*n* = 94, 3.4%). To visualize the relationships among these journals, we screened the journals on the basis of a minimum number of 11 related publications and constructed a journal network (Figure 5A). Figure 5A illustrates that *Journal of Alzheimer's Disease* had active citation relationships with *Stroke, Neurology, Acta Neuropathologica*, and *Neurobiology of Aging*, and others.

**Table 3:** Top 10 journals and co-cited journals on research of CAA.

**Rank**	**Journal**	**Count (%)**	**IF 2022**	**JCR 2022**	**Co-cited journal**	**Co-citation**	**IF 2022**	**JCR 2022**
1	Journal of Alzheimer's Disease	148 (5.3%)	4	Q2	Neurology	11,176	9.9	Q1
2	Stroke	141 (5.0%)	8.3	Q1	Stroke	8,891	8.3	Q1
3	Neurology	136 (4.9%)	9.9	Q1	Acta Neuropathologica	4,960	12.7	Q1
4	Acta Neuropathologica	94 (3.4%)	12.7	Q1	Annals of Neurology	4,685	11.2	Q1
5	Neurobiology of Aging	61 (2.2%)	4.2	Q2	Proceedings of the National Academy of Sciences of the United States of America	3,449	11.1	Q1
6	Journal of the Neurological Sciences	54 (1.9%)	4.4	Q2	Journal of Biological Chemistry	3,140	4.8	Q2
7	Acta Neuropathologica Communications	48 (1.7%)	7.1	Q1	Neurobiology of Aging	3,038	4.2	Q2
8	Journal of Stroke & Cerebrovascular Diseases	48 (1.7%)	2.5	Q3	Brain	2,700	14.5	Q1
9	Brain	46 (1.6%)	14.5	Q1	Journal of Neuroscience	2,693	5.3	Q1
10	Journal of Neuropathology and Experimental Neurology	44 (1.6%)	3.2	Q2	The Lancet Neurology	2,622	48	Q1

Among the 5,918 co-cited journals, 7 journals were co-cited more than 3,000 times, with *Neurology* (*n* = 11,176) being the most co-cited journal, prior to *Stroke* (*n* = 8,891), *Acta Neuropathologica* (*n* = 4,960), and *Annals of Neurology* (*n* = 4,685) (Table 3). In addition, we observed that 5 journals appeared on both the top 10 journals and top 10 co-cited journals lists. To build the co-citation network, we filtered the journals with a minimum co-citation count of 430 (Figure 5B). As depicted in Figure 5B, *Neurology* had positive co-citation relationships with *Stroke, Acta Neuropathologica, Annals of Neurology*, and *Proceedings of the National Academy of Sciences of the United States of America*, and others.

The citation relationships between journals and co-cited journals are visually represented through the dual-map overlay, with clusters of citing journals positioned on the left and clusters of cited journals positioned on the right (20). Figure 5C displays the main citation paths represented by the yellow and pink paths. These paths indicate that studies published in the fields of Molecular/Biology/Genetics were primarily cited by literature in the fields of Molecular/Biology/Immunology and Neurology/Sports/Ophthalmology journals.

### 3.5 Analysis of co-cited references

**Figure 6:**
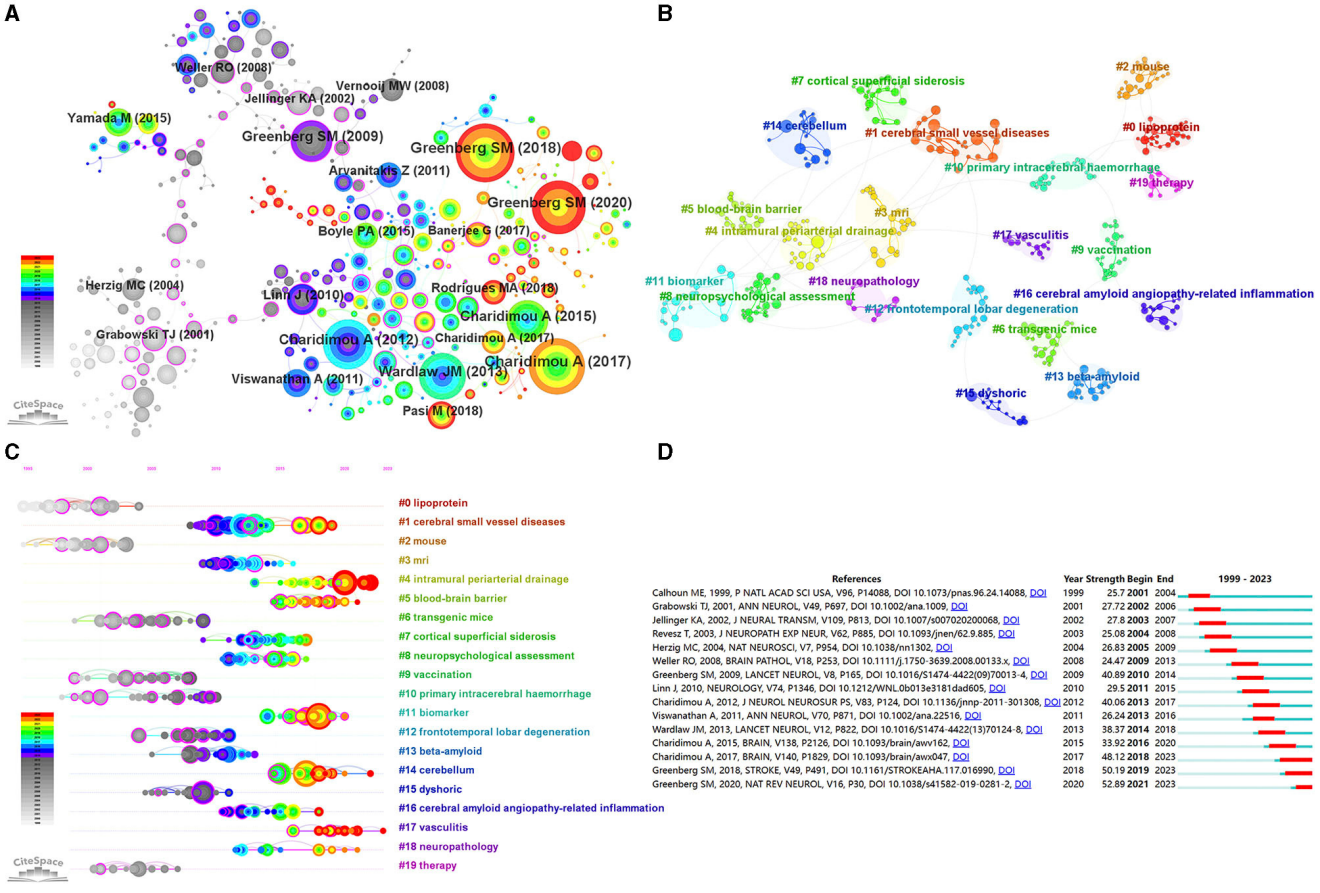
**(A)** The visualization network of co-cited references. **(B)** The cluster view of co-cited references. **(C)** The timeline view of the clusters. **(D)** Top 15 references with the strongest citation bursts.

To identify important papers in the field, we conducted an analysis of co-cited references with CiteSpace. The visualization network of co-cited references consisted of 387 nodes and 446 links (top 100 per slice, LBY = 5, *e* = 6.0; Figure 6A). Among the top 15 co-cited references (Table 4), all references were co-cited more than 50 times, with 4 references being co-cited over 100 times.

**Table 4:** Top 15 co-cited references on research of CAA.

**Rank**	**Title**	**DOI**	**Co-citation**	**Centrality**	**Year**
1	Diagnosis of cerebral amyloid angiopathy: evolution of the Boston criteria	doi: 10.1161/STROKEAHA.117.016990	125	0	2018
2	Emerging concepts in sporadic cerebral amyloid angiopathy	doi: 10.1093/brain/awx047	119	0.02	2017
3	Cerebral amyloid angiopathy and Alzheimer's disease - one peptide, two pathways	doi: 10.1038/s41582-019-0281-2	114	0.10	2020
4	Sporadic cerebral amyloid angiopathy revisited: recent insights into pathophysiology and clinical spectrum	doi: 10.1136/jnnp-2011-301308	106	0.01	2012
5	Neuroimaging standards for research into small vessel disease and its contribution to aging and neurodegeneration	doi: 10.1016/S1474-4422(13)70124-8	99	0.03	2013
6	Cortical superficial siderosis: detection and clinical significance in cerebral amyloid angiopathy and related conditions	doi: 10.1093/brain/awv162	88	0.09	2015
7	Cerebral microbleeds: a guide to detection and interpretation	doi: 10.1016/S1474-4422(09)70013-4	86	0.15	2009
8	Prevalence of superficial siderosis in patients with cerebral amyloid angiopathy	doi: 10.1212/WNL.0b013e3181dad605	64	0.16	2010
9	Cerebral amyloid angiopathy in the elderly	doi: 10.1002/ana.22516	59	0	2011
10	Mixed-location cerebral hemorrhage/microbleeds: underlying microangiopathy and recurrence risk	doi: 10.1212/WNL.0000000000004797	59	0	2018
11	Cerebral amyloid angiopathy: emerging concepts	doi: 10.5853/jos.2015.17.1.17	59	0	2015
12	Cerebral amyloid angiopathy and cognitive outcomes in community-based older persons	doi: 10.1212/WNL.0000000000002175	54	0	2015
13	Cerebral amyloid angiopathy pathology and cognitive domains in older persons	doi: 10.1002/ana.22112	53	0.02	2011
14	The Edinburgh CT and genetic diagnostic criteria for lobar intracerebral hemorrhage associated with cerebral amyloid angiopathy: model development and diagnostic test accuracy study	doi: 10.1016/S1474-4422(18)30006-1	52	0.05	2018
15	The increasing impact of cerebral amyloid angiopathy: essential new insights for clinical practice	doi: 10.1136/jnnp-2016-314697	50	0.11	2017

It is important to note that the effectiveness of mapping can be evaluated using two important parameters: the modularity value (*Q*-value) and the mean silhouette value (*S*-value). A *Q*-value greater than 0.3 and an *S*-value greater than 0.7 indicate significant clustering. As shown in Figure 6B and Supplementary Table 1, cluster analysis revealed a total of 20 clusters with a *Q*-value of 0.8748 and a mean *S*-value of 0.9629, indicating the credibility of the clustering results. These clusters primarily included “lipoprotein” (#0), “cerebral small vessel diseases” (#1), “mouse” (#2), “mri” (#3), “intramural periarterial drainage” (#4), and “blood-brain barrier” (#5) (Figure 6B). Furthermore, the timeline view of the clusters reflected the research hotspots. Specifically, the clusters “intramural periarterial drainage” (#4), “blood-brain barrier” (#5), “biomarker” (#11) “cerebellum” (#14), “vasculitis” (#17), and “neuropathology” (#18) emerged as hotspots in recent years (Figure 6C).

The analysis of citation bursts allows us to identify studies that have garnered significant attention from researchers in the same field and to identify studies that will have a substantial impact on future investigations. Figure 6D presents the top 15 references with the strongest citation bursts. The first reference with a citation burst was entitled “Neuronal overexpression of mutant amyloid precursor protein results in prominent deposition of cerebrovascular amyloid” and was published in 2001 (21). The reference entitled “Cerebral amyloid angiopathy and Alzheimer's disease - one peptide, two pathways” had the highest citation burst value (strength = 52.89) during the period of 2021–2023 (22). In general, the burst strength of the top 15 references varied between 24.47 and 52.89.

### 3.6 Analysis of keywords

**Figure 7:**
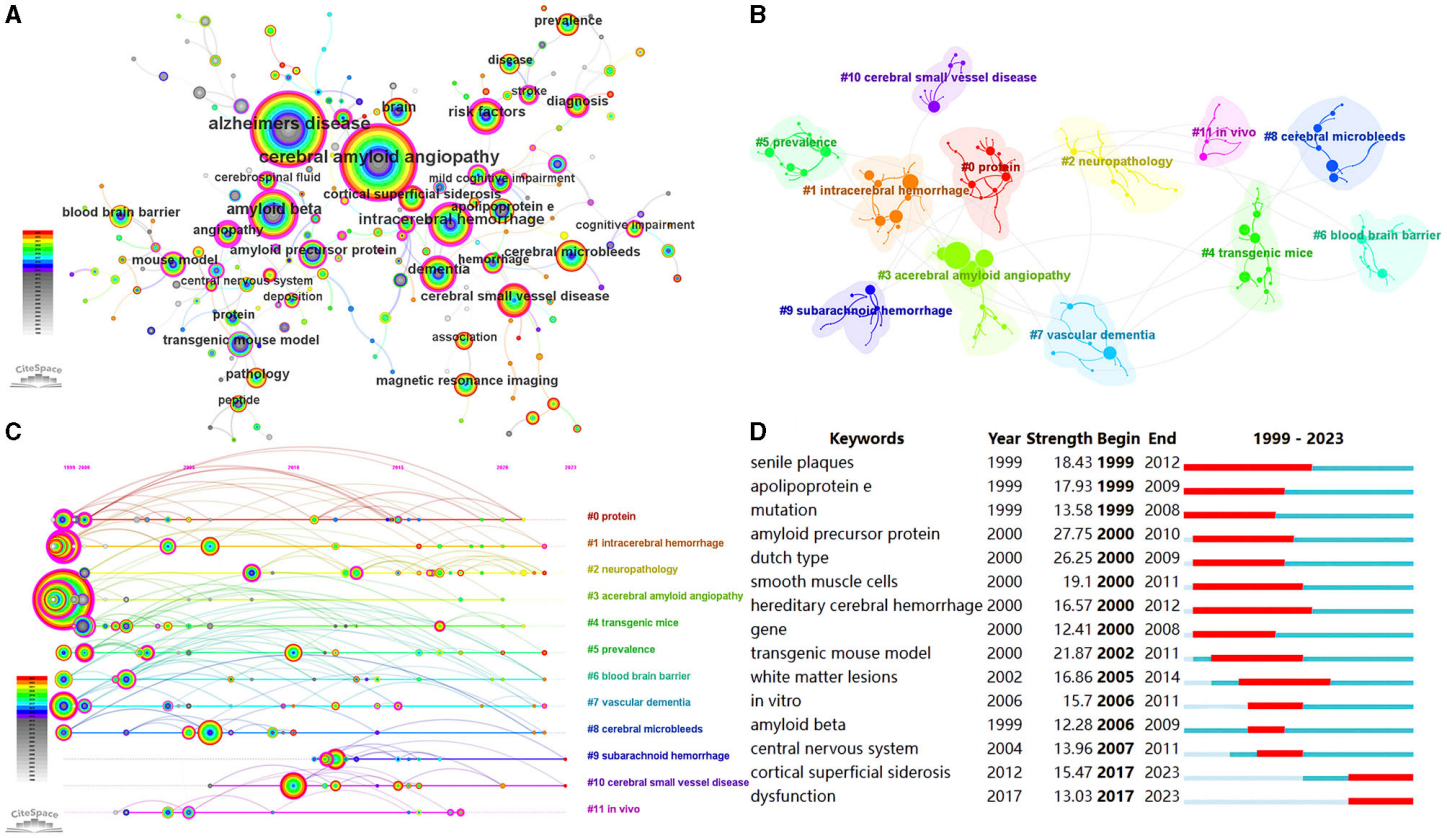
**(A)** The visualization network of keywords. **(B)** The cluster view of keywords. **(C)** The timeline view of the clusters. **(D)** Top 15 keywords with the strongest citation bursts.

The analysis of keywords provides valuable insights into the research focus of an article or author, offering a comprehensive overview of research trends. In this study, we utilized pathfinder, pruning sliced networks, and pruning the merged network to analyze the co-occurrence network map of keywords (top 150 per slice, *e* = 4.0), resulting in 192 nodes and 224 links (Figure 7A). Table 5 presents the top 20 keywords related to CAA research. Apart from the keywords “cerebral amyloid angiopathy” and “angiopathy”, other frequently occurring keywords were strongly associated with various aspects of CAA research. Notably, 13 of these keywords exhibited a centrality above 0.10, underscoring their significance in the field of CAA.

**Table 5:** Top 20 keywords on research of CAA.

**Rank**	**Keyword**	**Count**	**Centrality**
1	cerebral amyloid angiopathy	1,569	0.28
2	alzheimer's disease	1,486	0.38
3	amyloid beta	690	0.54
4	intracerebral hemorrhage	531	0.57
5	dementia	385	0.34
6	risk factors	376	0.43
7	brain	366	0.04
8	cerebral microbleeds	306	0.07
9	cerebral small vessel disease	305	0.18
10	amyloid precursor protein	279	0.34
11	pathology	266	0
12	diagnosis	260	0.22
13	apolipoprotein e	235	0.09
14	transgenic mouse model	232	0.17
15	blood brain barrier	223	0.08
16	magnetic resonance imaging	213	0.02
17	cortical superficial siderosis	202	0.19
18	mouse model	201	0.26
19	prevalence	198	0.03
20	angiopathy	193	0.61

As shown in Figure 7B and Supplementary Table 2, we obtained 12 clusters in total: “protein” (#0), “intracerebral hemorrhage” (#1), “neuropathology” (#2), “cerebral amyloid angiopathy” (#3), “transgenic mice” (#4), “prevalence” (#5), “blood brain barrier” (#6), “vascular dementia” (#7), “cerebral microbleeds” (#8), “subarachnoid hemorrhage” (#9), “cerebral small vessel disease” (#10), and “*in vivo*” (#11). The *Q*-value was 0.8097 and the mean *S*-value was 0.9465, indicating the effectiveness and homogeneity of these clusters. The cluster names were refined based on the keywords within each cluster. Furthermore, in Figure 7C, we presented a timeline view of the clusters, enabling an understanding of the evolutionary characteristics of each cluster over time. It can be seen that the cluster “subarachnoid hemorrhage” (#9), “cerebral small vessel disease” (#10), and “*in vivo*” (#11) emerged relatively later compared to other clusters.

Keywords bursts refer to frequently occurring keywords within a specific period of time, allowing for the tracking of research hotspots. As shown in Figure 7D, “amyloid precursor protein” exhibited the strongest burst (strength = 27.75), followed by “dutch type” (strength = 26.25) and “transgenic mouse model” (strength = 21.87). Over the 25-year observation period, the research foci predominantly centered around the pathogenesis of CAA. In recent years, “cortical superficial siderosis” and “dysfunction” have emerged as primary research hotspots, suggesting potential future research directions for CAA.

## 4 Discussion

This scientometric analysis study examined the research development of CAA over the past 25 years. We utilized VOSviewer and CiteSpace to analyze 2,798 papers on CAA research from SCI-E, WoSCC. The annual number of publications ranged from 43 to 200 between 1999 and 2023, demonstrating an overall upward trend with slight fluctuations. This increased interest in the field may be attributed to the growing importance and global burden of stroke (23).

The distribution of CAA research output among the top 10 productive countries/regions, with a majority being high-income, reflects the influence of economic status and governmental healthcare expenditure on medical research productivity. Notably, the United States, with the largest GDP, emerged as the leading country in CAA publications, which can be partially attributed to its substantial healthcare investment and robust research infrastructure. Furthermore, collaborations in CAA research were predominantly centered around the United States, underscoring its pivotal role in advancing this academic field. This observation emphasizes the necessity for enhancing collaborations among other countries/regions to further advance CAA research globally. Similarly, numerous institutions in the United States, England, and the Netherlands extensively published on CAA. Among the top 10 leading institutions, 6 were distributed in the United States. Interestingly, despite Japan being the third most productive country/region, no Japanese institution ranked among the top 10, indicating potential areas for growth and collaboration.

Authorship analysis revealed that Greenberg from Massachusetts General Hospital made the greatest contributions with 173 publications and 1,845 co-citations. His latest research identified a link between chronic cortical iron deposition resulting from cSS and local reactive astrogliosis in CAA, suggesting potential damaging effects of iron deposition on cortical parenchyma (24). Charidimou made the second greatest contributions with 109 publications and 1,788 co-citations. Charidimou used to work together as colleagues with Greenberg. The most co-cited reference entitled “Diagnosis of cerebral amyloid angiopathy: evolution of the Boston criteria” was co-authored by them (6). Furthermore, author collaboration and co-citation networks centered around Greenberg and Charidimou confirmed their influential roles in the field.

The quality and prestige of journals are crucial for disseminating research findings. Journal IF serves as a significant metric for assessing the quality of a journal by measuring its citation frequency over time. Journal Citation Report (JCR) is extensively utilized for assessing the worldwide influence of journals, categorizing them into quartiles based on their IF rank, with Q1 representing the most influential and Q4 representing the least influential. Within the ranks of the top 10 journals with the highest productivity in the field of CAA, most relevant studies were published in Q1 or Q2 journals with IF greater than 3, except *Journal of Stroke & Cerebrovascular Diseases*. These journals held a significant position in this field. In terms of the top 10 co-cited journals, all journals were in Q1 or Q2. Notably, *Neurology, Stroke, Acta Neuropathologica, Neurobiology of Aging*, and *Brain* appeared in both the lists of top 10 journals and top 10 co-cited journals, reflecting their significant influences. Research findings related to CAA published in these journals are likely to receive greater attention.

The frequency of co-citations, analyzed using CiteSpace, can provide insights into the influence of papers within a specific research field. Among the top 15 co-cited references on CAA research, 9 were literature reviews that comprehensively summarized the progress in CAA research from various perspectives, including epidemiology, pathology and pathophysiology, clinical manifestations, imaging features, diagnostic criteria, risk factors, and treatment (1, 2, 4, 6, 22, 25–28). Within the remaining 6 original articles, 3 focused on the imaging characteristics of MRI or CT and their role in diagnosing CAA (29–31), 2 articles published by Schneider and colleagues were related to the cognitive outcomes in CAA patients (32, 33), and the remaining article evaluated potential mechanisms and the risk of recurrence of ICH in patients with ICH/microbleeds in both lobar and deep hemispheric brain regions (34). The keywords co-occurrence analysis provides insights into the prevalence and interconnectedness of research topics within the scientific community. Besides the terms “cerebral amyloid angiopathy” and “angiopathy”, other frequently used keywords primarily focused on the diagnosis and pathogenesis of CAA. Notably, the keyword “Alzheimer's disease” appeared frequently. The reasons may be that CAA and Alzheimer's disease often overlap and the pathogenic mechanisms of the both conditions converge at various stages, such as Aβ production and circulation, and its clearance from the brain (22).

The co-cited references and keywords co-occurrence clustering helped categorize the entire network into distinct clusters, each representing a primary topic. The cluster labels “transgenic mice”, “blood brain barrier”, “cerebral small vessel disease”, and “neuropathology” appeared in both the cluster analysis of co-cited references and keywords co-occurrence. Animal models, particularly transgenic mouse models, are indispensable in investigating the mechanisms and potential therapies for CAA. Since rodents do not naturally develop CAA, various transgenic mouse models, such as APPDutch mice, APP23 mice, Tg2576 mice, and PDAPP mice, have been developed to mimic cerebral amyloidosis seen in humans (35, 36). In addition, the APPDutch mice are the unique murine model that develops significant Aβ-CAA without the presence of parenchymal amyloid plaques (37). The blood brain barrier (BBB) is a critical structure involved in the transport of Aβ and is in close relation to the occurrence and progression of CAA (38). The Receptor for Advanced Glycation Endproducts is the primary transporter responsible for the entry of Aβ into the brain parenchyma through the BBB (39). While the efflux process is mainly mediated by the transporters low-density lipoprotein receptor-related protein 1 and P-glycoprotein1 (40, 41). Alterations in the expression of these transport proteins can impact Aβ transport, leading to its deposition on blood vessel walls, which is age-related (38). The cluster labels “primary intracerebral hemorrhage”, “intracerebral hemorrhage”, “cerebral microbleeds”, and “subarachnoid hemorrhage” as hemorrhagic manifestations of CAA are noteworthy, attributed to the gradual vascular deposition of Aβ and decline of vascular smooth muscle cells. CAA ranks as the second most frequent cause of ICH in individuals over 60 years old (42), significantly associated with lobar ICH, but not with deep ICH (43), due to the locations of affected vessels. The annual incidence of CAA-related ICH recurrence is higher than that of CAA-unrelated ICH (44). Hemorrhagic manifestations may not always be evident clinically, as observed with CMBs, which were identified in nearly half of the CAA patients (29). Patients with lobar CMBs face a significant risk of experiencing lobar ICH in the future (45). In addition, CAA may manifest as cSAH, stemming from CAA involvement in leptomeningeal vessels or extending from a lobar ICH.

The burst detection analysis is a valuable method for investigating the evolution of research hotspots within an academic field. Articles or keywords that experience high citation bursts indicate active discussion or usage during a specific period. Among the top 15 references with the strongest citation bursts, the 9 references with the most recent burst begin times overlap with the top 9 co-cited references in CAA research, highlighting the importance of these papers in this field (1, 4, 6, 22, 25–27, 29, 30). The keyword “cortical superficial siderosis” has been an ongoing burst keyword since 2017. It refers to the deposition of hemosiderin, a breakdown product of blood, in a linear pattern within the subarachnoid space, leptomeninges, and superficial layers of the cerebral cortices (46). This deposition is restricted to the supratentorial compartment and the convexities of the cerebral hemispheres (46). cSS is a key feature of CAA and has emerged as a diagnostic indicator in the modified Boston criteria (v1.5) for CAA (6). cSS is related to transient focal neurological episodes and may serve as a marker for future risk of ICH in CAA patients (27, 47, 48). Research on cSS continues to be a hot topic. Van Harten et al. proposed a semi-automated method for quantifying cSS burden on MRI, which may be valuable for further studies in CAA cohorts (49). Tanaka et al. investigated the association between post-stroke epilepsy and cSS, revealing that cSS may contribute to the development of post-stroke epilepsy (50). Considering the role of cSS in CAA progression and related complications, the development of effective treatments to regulate cortical iron deposits may be a future research direction.

The development and utilization diagnostic tools for CAA has been a long-standing research focus in the field. The gold standard for definitely diagnosing CAA currently relies on histopathological confirmation, which is only feasible in patients with available brain tissue. Nevertheless, there are several clinical-neuroimaging criteria that can identify potential cases. The most widely used of these criteria are the MRI-based Boston criteria, which defined probable or possible CAA based on clinical and MRI information. The original Boston criteria (v1.0) in 1995 defined probable CAA as having at least two hemorrhagic lesions confined to lobar brain regions (51). In the modified Boston criteria (v1.5) put forward in 2010, cSS was added as an additional hemorrhagic lesion, increasing sensitivity without affecting specificity (29). The most recent Boston criteria (v2.0) expanded the diagnosis of probable and possible CAA to include severe perivascular spaces in the centrum semiovale and WMHs in a multispot pattern, further enhancing sensitivity without compromising specificity (52). Additionally, in 2018, the CT-based Edinburgh criteria were proposed as an alternative, particularly for patients with lobar ICH who are unable to undergo MRI examination (31). Novel imaging techniques allow for *in vivo* analysis of vascular changes in physiology and pathology. Dysfunctional cerebrovascular reactivity, assessed through blood-oxygen-level dependent functional MRI response to visual stimulation, has been recognized as a characteristic of CAA (53). Amyloid-PET in CAA has been conducted using ^18^F-florbetapir or ^11^C-Pittsburgh compound B, both of which specifically target Aβ (54). However, the clinical utility of these modern tools for CAA diagnosis is currently limited and remains under investigation. Research on the combined use of these novel technologies with validated diagnostic criteria for CAA holds promise for further advancements in CAA research and diagnosis.

As a scientometric analysis, this study is subject to several limitations. Firstly, all data were retrieved and downloaded from WoSCC. Even though this database is considered the most suitable database for scientometric analysis, it may have missed a few relevant studies not included in this database. Secondly, only studies published in English were included, potentially underestimating the impact of non-English publications. Thirdly, the major limitations of scientometric analysis of keywords are inconsistency in keyword usage and subjectivity in keyword selection. Authors may use different keywords to describe the same concept, and keywords are assigned based on their own judgment and understanding, which may result in potential biases. Lastly, it is important to note that the analysis of highly co-cited papers may overlook recently published papers with significant contributions due to their lower citation rates. This is a form of bias as these recent papers have had less time to accumulate citations. This underscores the need for future updates to continually assess the impact of these recent papers, as they might have a higher impact in a few years.

## 5 Conclusion

This scientometric analysis examined the research history of CAA over the past 25 years with scientometric software. A total of 2,798 relevant papers were retrieved from SCI-E, WoSCC. The findings revealed a general increase in the number of published studies on CAA. The United States was the core country with the highest number of publications. It dominated the field and formed a network of academic collaborations with numerous countries/regions. Additionally, we identified key institutions, scholars, and journals that had significant influences in this field. Finally, we analyzed the references and keywords, to provide scientific insights for researchers and clinicians. In summary, this study offers a comprehensive overview for scholars to understand the current state and trends in CAA research, serving as a valuable reference for future related research.

## Data availability statement

The original contributions presented in the study are included in the article/Supplementary material, further inquiries can be directed to the corresponding author.

## Author contributions

KW: Formal analysis, Writing—original draft. BZ: Conceptualization, Supervision, Writing—review & editing. HDu: Writing—review & editing. HDuan: Writing—review & editing. ZJ: Writing—review & editing. SF: Conceptualization, Funding acquisition, Supervision, Writing—review & editing.
