# Supplementary material for: Research landscape and trends of cerebral amyloid angiopathy: a 25-year scientometric analysis
Source: Front Neurol. 2024 Jan 8;14:1334360. doi: 10.3389/fneur.2023.1334360 (PMC10800472; doi:10.3389/fneur.2023.1334360)
Supplement: Supplementary file 1 [file Table_1.DOCX]

Supplementary Material

**Supplementary Table 1** Cluster analysis of co-cited references on research of CAA

| **Cluster ID** | **Size** | **Silhouette** | **Mean year** | **Label** |
| --- | --- | --- | --- | --- |
| #0 | 24 | 0.937 | 1999 | lipoprotein |
| #1 | 24 | 0.920 | 2012 | cerebral small vessel diseases |
| #2 | 24 | 0.984 | 1999 | mouse |
| #3 | 24 | 0.978 | 2012 | mri |
| #4 | 23 | 0.979 | 2017 | intramural periarterial drainage |
| #5 | 23 | 0.991 | 2017 | blood-brain barrier |
| #6 | 22 | 0.986 | 2004 | transgenic mice |
| #7 | 22 | 0.991 | 2014 | cortical superficial siderosis |
| #8 | 21 | 0.936 | 2013 | neuropsychological assessment |
| #9 | 20 | 0.954 | 2004 | vaccination |
| #10 | 20 | 0.945 | 2004 | primary intracerebral haemorrhage |
| #11 | 20 | 0.930 | 2017 | biomarker |
| #12 | 19 | 0.884 | 2008 | frontotemporal lobar degeneration |
| #13 | 18 | 1.000 | 2009 | beta-amyloid |
| #14 | 16 | 0.956 | 2018 | cerebellum |
| #15 | 15 | 0.981 | 2007 | dyshoric |
| #16 | 14 | 1.000 | 2013 | cerebral amyloid angiopathy-related inflammation |
| #17 | 14 | 0.953 | 2020 | vasculitis |
| #18 | 13 | 1.000 | 2015 | neuropathology |
| #19 | 11 | 0.980 | 2003 | therapy |

**Supplementary Table 2** Cluster analysis of keywords on research of CAA

| **Cluster ID** | **Size** | **Silhouette** | **Mean year** | **Label** |
| --- | --- | --- | --- | --- |
| #0 | 23 | 0.960 | 2008 | protein |
| #1 | 22 | 0.956 | 2007 | intracerebral hemorrhage |
| #2 | 19 | 1.000 | 2016 | neuropathology |
| #3 | 18 | 1.000 | 2002 | cerebral amyloid angiopathy |
| #4 | 18 | 0.889 | 2006 | transgenic mice |
| #5 | 16 | 0.956 | 2009 | prevalence |
| #6 | 16 | 0.988 | 2012 | blood brain barrier |
| #7 | 15 | 0.780 | 2009 | vascular dementia |
| #8 | 14 | 0.973 | 2012 | cerebral microbleeds |
| #9 | 12 | 0.937 | 2014 | subarachnoid hemorrhage |
| #10 | 10 | 0.987 | 2014 | cerebral small vessel disease |
| #11 | 9 | 0.898 | 2010 | in vivo |
